# Prevalence of brucellosis among patients attending Wau Hospital, South Sudan

**DOI:** 10.1371/journal.pone.0199315

**Published:** 2018-06-27

**Authors:** Nuol Aywel Madut, George William Nasinyama, John Bwalya Muma, Kenneth L. L. Sube, Moses Ocan, Adrian Muwonge, Jacques Godfroid, Ambrose Samuel Jubara, Clovice Kankya

**Affiliations:** 1 Department of Clinical Studies, Faculty of Veterinary Science, University of Bahr el Ghazal, Wau, South Sudan; 2 Department of Biosecurity, Ecosystems &Veterinary Public Health (BEP), College of Vet. Animal Resources & Biosecurity (COVAB), Makerere University, Kampala, Uganda; 3 Department of Disease Control, School of Veterinary Medicine, University of Zambia, Lusaka, Zambia; 4 Department of Biochemistry, College of Medicine, University of Juba, Juba, South Sudan; 5 Department of Pharmacology & Therapeutics, College of Health Sciences, Makerere University, Kampala, Uganda; 6 Department of Genetics and Genomics, The Roslin Institute, Royal (Dick) School of Veterinary Studies, University of Edinburgh, Easter Bush, Midlothian, United Kingdom; 7 Department of Arctic and Marine Biology, University of Tromsø-the Arctic Univertsity of Norway, Faculty of Biosciences, Fisheries and Economics, Tromsø, Norway; University of Minnesota, UNITED STATES

## Abstract

Brucellosis is a zoonotic disease of public health importance; its prevalence varies globally. In low-income countries, brucellosis is an endemic and neglected disease affecting both animals and humans. This study was intended to establish brucellosis sero-prevalence among patients attending Wau hospital, South Sudan. Across sectional study, was done among randomly selected patients attending Wau hospital. Data was collected using questionnaires and laboratory investigations. Rose Bengal plate Test (RBPT), Serum agglutination test (SAT) and Competitive Enzyme Linked Immuno Sorbent Assay (c-ELISA) was used in the analysis of blood samples serially starting with RBPT which is more sensitive and least specific then SAT. c-ELISA test which is most specific and less sensitive compared to RBPT and SAT was then used to confirm presence of Brucella antibodies in the samples. A total of 416 participants out of 1664 were enrolled to this study. The majority of participants were between 7-to-76 years of age with mean age of 30.72 (SD+/- 12.83). The sero-positivity of patient’s blood samples for brucellosis using c-ELISA was 23.3% (97/416) among patients presenting to Wau hospital. Socio-demographic characteristics, occupation, clinical signs of disease and types of animals reared by animal owners showed no significant correlation with occurrence of sero-positivity among patient’s blood samples for brucellosis. While ethnicity (Nilotic), knowledge of zoonotic disease, and consumption of animal urine were statistically significant (p<0.05). The study found a high prevalence of brucellosis among febrile patients attending Wau hospital general outpatient clinic. There is need for co-ordination and collaboration between veterinary and health sectors of government to help prevent and control brucellosis in the region.

## Introduction

Globally, brucellosis is considered to be the most common zoonotic disease, with more than 500,000 cases recorded yearly [1]. Furthermore, it is of public health and economic burden to livestock production systems especially in pastoral and agro-pastoral communities [2]. The economic loss is mainly due to abortions, giving birth to weak calves and decrease in milk productivity in addition to posing a major obstacle for international trade.

Inadequate preventive and control measures potentially influence disease transmission between animals and humans in the community [3, 4]. Brucellosis is an occupational hazard among herders, veterinarians, laboratory technicians, butchers, and handlers of infected animal products. In addition, the disease is common among community members who consume poorly prepared animal products such as meat and milk. Human brucellosis has a broad clinical picture as its presentation mimics conditions like malaria and typhoid fever, joint diseases and other conditions causing pyrexia [3, 5]. This usually causes diagnostic challenges for brucellosis in health facilities especially in developing countries due to inadequate laboratory facilities. The disease manifests with intermittent or irregular fever, headache, weakness, profuse sweating, chills, arthralgia, depression, weight loss, and generalized aching.

In sub-Saharan Africa, the prevalence of brucellosis is not clear with reports varying from country to country and the disease has been reported in most parts of Africa [3, 6]. This variation could be attributed to diagnostic challenges, underreporting, and lack of surveillance systems [7]. In most low and middle income countries where Brucellosis is endemic, physicians diagnose disease using clinical symptoms due to inadequate laboratory facilities. However, Brucellosis shares clinical symptoms with other diseases like tuberculosis and malaria common in these countries. This increases the risk of misdiagnosis and treatment of the disease and potentially worsening of disease outcomes. The prevalence of Brucellosis in most communities of Africa is not known. Therefore this study was intended to establish the prevalence and factors associated with occurrence of brucellosis among febrile patience’s attending Wau regional referral hospital in South Sudan.

## Material and methods

### Study area and setting

The study was conducted in Wau municipality, Wau state located in Bahr el Ghazal region northwestern South Sudan. Wau state is approximately 650 kilometers (400 miles) northwest of Juba, the capital city. Data were collected among patients at Wau regional referral hospital. The hospital provides both general and specialized health care services to the population.

### Study population

This study included all patients presenting to the outpatient department of Wau regional referral hospital. Data were collected from December 2015 to May 2016.

### Study design and sample size

This was a cross sectional study. The Sample size was estimated using a standard formulae for cross-sectional studies Trusfield (1995)[8].

The expected prevalence of Brucellosis was assumed to be 50% and 95% level of significance, Z value of 1.96, q = (1-P), and d = 5%.

$$N=\frac{Z^{2}PQ}{d^{2}}$$

$$N=\frac{1.96^{2}*0.5*0.5}{0.05^{2}}$$

N = 384 samples, therefore the calculated study sample size was 384.

### Sampling procedure

**Fig 1:**
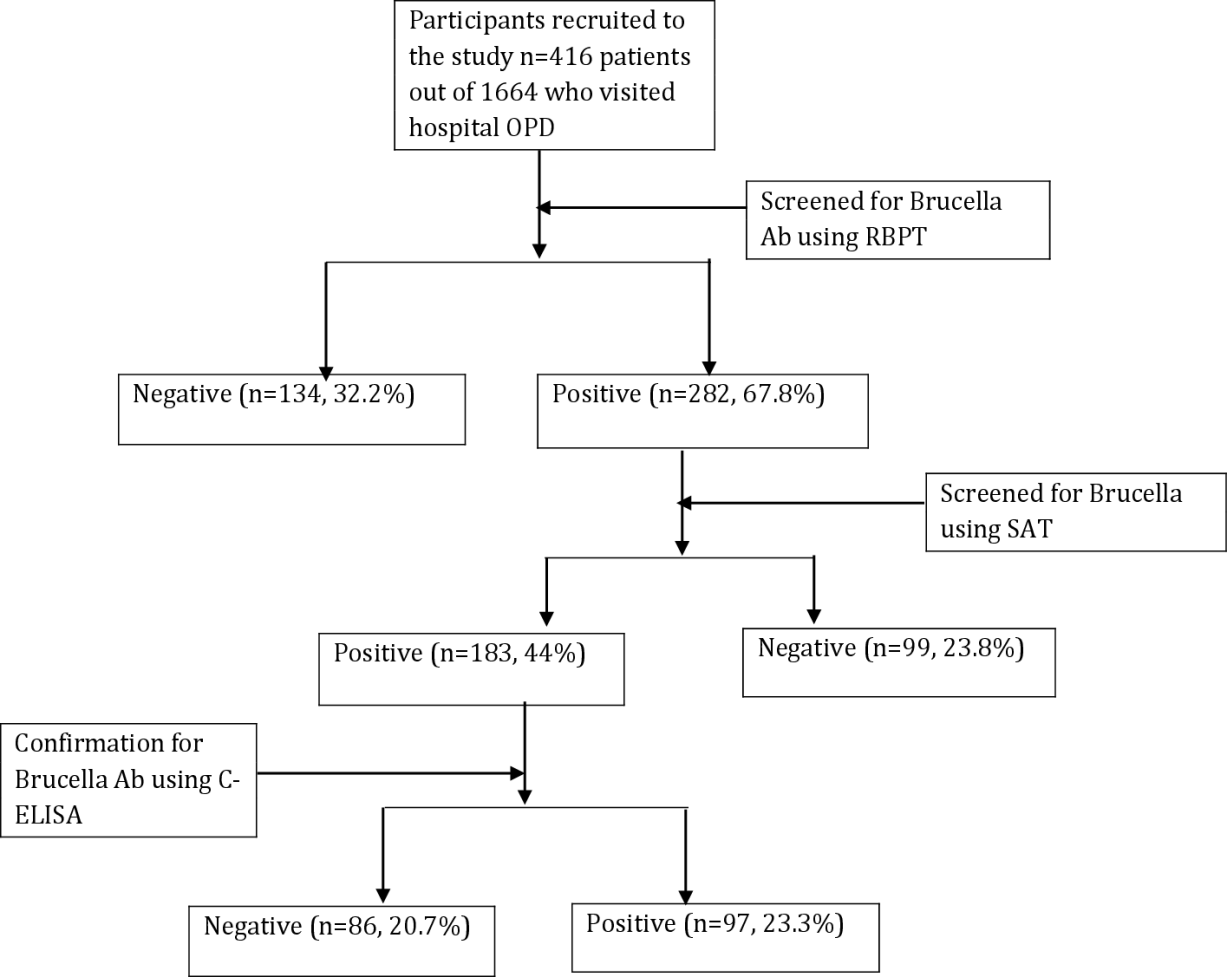
Summary of the outcomes of patient recruitment in the study.

The patients, 1664 who presented to the outpatient department of Wau regional referral hospital from December 2015 –to—May 2016 were recruited into the study using systematic random sampling. Every fourth (4^th^) patient who was waiting to see the clinician was contacted for recruitment into the study. The patients were first briefed about the study before obtaining a written informed consent. Data on socio-demographic characteristics, knowledge on zoonotic diseases and previous medical history was collected using an interview-administered questionnaire. After the interview each of the study participants were then referred to the laboratory for blood sample collection. From each of the patients, 5ml of venous blood (cephalic vein) was collected into plain vacutainer tube for serological examination. The blood samples were serially screened for Brucella antibodies using RBPT then SAT and the diagnosis of Brucellosis confirmed using c-ELISA (Fig 1).

### Sample processing and analysis

The blood samples in vacutainer tubes were centrifuged immediately after collection at 1500 rpm for ten (10) minutes. After centrifugation, serum was aspirated into separate Eppendorf tubes and then stored at -80°C at Wau hospital laboratory until analysis. The samples were transferred in an ice bag to Makerere University, College of Veterinary Science and Biosecurity (COVAB) central laboratory for serological analysis. The samples were thawed and left to stand for 15 minutes at room temperature at COVAB central laboratory. The Eppendorf tubes containing serum were then centrifuged at 1500 rpm for 10 minutes. Part of the supernatant was aspirated using a Pasteur pipette and used for serological screening and the other portion transferred into cryovials and stored at -80°C for future analysis.

### Serological screening

Two tests namely Rose Bengal Plate test (RBPT) and Standard Tube Agglutination Test (SAT) were used for screening for Brucella antibodies in the samples. Presence of Brucella antibodies (IgG) in the samples was then confirmed using Competitive Enzyme Immuno Sorbent Assay (C-ELISA). All samples (416) were first screened for Brucella antibodies using RBPT the samples that turned positive were further screened using SAT. The samples that were positive in both tests were then confirmed to have Brucella antibodies using c-ELISA. The tests are briefly described below however, the details of the test procedures for RBPT, SAT and c-ELISA are provided in S1 Serological tests.

#### Rose bengal plat test (RBPT)

The samples were screened using RBPT for *B*. *abortus* and *B*. *melitensis* antigens according to the manufacturer’s instructions. The antigen reagent was kindly provided from Veterinary Research Institute (VRI) at Soba, Sudan. Briefly, a drop of serum was placed on clean glass slide and a drop of Brucella antigen (M) for *Brucella melitensis* was added. Onto a second slide a drop of serum was placed and then a drop of *Brucella abortus* antigen (A) was added. The slide was then rotated gently to mix the sample on the slide. Agglutination occurred within 10 to 15 minutes in the samples positive for Brucella antibodies. The negative samples showed no agglutination. Internal positive and negative controls were tested alongside the patient samples for quality assurance. All positive sera detected with rapid antigen test were subsequently tested using Standard Tube Agglutination Test.

#### Standard tube agglutination test (SAT)

Is a principle serological test used to detect brucellosis. It detects agglutinating antibodies of the IgM, IgG1, IgG2, and IgA types. The SAT is relatively simple and easy to perform but it requires basic laboratory equipment. Provided by Central Diagnostic Laboratory, (JICA) Makerere University a titre of ≥1: 80 were considered as positive. 50 microliters of serum was diluted with 950μL normal saline (1:20) and 500 μL from the diluted serum was added in to another tube with 500 μL from brucella antigen (1000 μL). 500-μL was then transferred and added into another 500 μL and incubated at 37^**o**^ C for 24 hours. The positive results showed agglutination and the negative results do not show any agglutination.

#### Competitive enzyme linked immune-sorbent assay (C-ELISA)

Brucellosis was screened in the samples using the IDEXX Brucellosis Serum X2 Antibody (Ab) test kit. IDEXX is an immunological (ELISA) test kit containing immunoglobulin IgG and IgM with Anti-ruminant IgG and IgM HRPO conjugate (IDEXX Montpellier SAS, France). It is used for detection of antibodies against *Brucella abortus* and *Brucella melitensis* in individual and pooled serum. The c-ELISA test was performed following a modified manufacturer’s procedure [9, 10].

#### Interpretation of test results for c-ELISA

The results were interpreted as follows: samples with S/P (sample to positive) percentage less than or equal to 110% were considered negative for brucella antibodies. Samples with S/P percentage greater 110% and less than 120% were considered suspect. Samples with S/P percentage greater than or equal to 120% were considered positive for the presence of Brucella antibodies [9, 10].

#### Quality control for the serological tests

Positive and negative control sera were run in parallel with each test. Duplicates of each tested serum were used to assure that the antigens used in the test were sensitive as well as specific [11].

### Data analysis

Data was entered, double-checked and cleaned prior to the analysis (S1 Serological tests). Data was analyzed using SPSS version 24. Descriptive and analytical statistics were used to summarize the data obtained. Odds ratio and chi-square tests were used to compare variables. Results were considered as significant if the p-value was < 0.05. It was not possible to use sensitivity and specificity for evaluation of serological tests in this study because the gold standard was absent. The variables that showed significance, P = 0.05 at bivariate analysis were included in multivariable analysis using backward elimination method. Model fitness was tested using Hosmer-Lemeshow test for goodness-of-fit, which passed with a p-value of 0.91.

### Ethical approval and consent to participant

The study protocol (SBLS/REC/15/133), was assessed and approved by the Ethical Review Committee of the College of veterinary medicine, Animal Resources and Biosecurity (COVAB), Makerere University, Kampala, Uganda the reference number of SBLS. NA. 2015 (S1 Ethical Approval). The National Ministry of Health, Juba South Sudan (S2 Ethical Approval). The study objective was explained to participants and guardians of the minors where informed written consent was obtained from the study participants and guardians of the minors who had agreed to participate in the study (S3 Ethical Approval). Each participant was interviewed independently and the collected data was kept confidential. Study numbers were used instead of participants’ names to ensure confidentiality. Moreover, import and export permits of the biological samples were obtained from Ministry of Agriculture, Animal Industry and Fisheries (MAAIF)—LHE 46/172/406, Uganda (S4 Ethical Approval), Ministries of Health (MOH), and Livestock and Fisheries Industry (MLFI), South Sudan—RSS/MLFI/DVS/J/39 (S5 Ethical Approval), respectively, prior to shipment from and to designate country.

## Results

### Socio-demographic characteristics

A total of 416 patients who visited Wau referral hospital from December 2015-to- May 2016 were recruited and screened during the study. Half, 50.7% (211/416) of the study participants were females. The age range of the study participants was 7–76 years with the mean of 30.7±12.8. The majority, 65.4% (272/416) of the patients were aged 16–35 years. Over a third, 33.2% (138/416) of the patients had never gone to school at all (Illiterate). Over a third, 43.0% (179/416) of the study participants were of Nilotic origin (Table 1).

**Table 1:** Socio-demographic characteristics of the study participants.

Characteristic	Level	Frequencies (N = 416)	Percentages (%)
Sex	Female	211	50.7
	Male	205	49.3
Age Range	5–15	26	6.3
(Years)	16–35	272	65.4
	36–60	107	25.7
	>60	11	2.6
Marital Status	Cohabiting	2	0.5
	Married	256	61.5
	Single	160	38.5
Education level	Primary	72	17.3
	Intermediate	77	18.5
	Secondary	63	15.1
	Higher Education	66	15.9
	Illiterate	138	33.2
Religion	Christian	356	85.6
	Muslim	56	13.5
	Others	4	1.0
Occupation	Butcher	3	0.7
	Business/Trader	15	3.6
	Nurse/Midwife	4	1.0
	Housewife	35	8.4
	Student	120	28.8
	Restaurant worker	29	7.0
	Veterinary	5	1.2
	Farmer	9	2.2
	Jobless	40	9.6
	Others	156	37.5
Ethnic Grouping	Nilotic	179	43.0
	Lou	47	11.3
	Equatorian	19	4.6
	Bantu	93	22.4
	Arab	24	5.8
	Fur	42	10.1
	Falata	8	1.9
	Others	4	1.0
Knowledge about zoonotic diseases	No	371	89.2
	Yes	45	10.8

#### Prevalence of brucellosis

The study found Brucellosis prevalence of 23.3% (97/416) among febrile patients attending outpatients departments of Wau referral hospital (Fig 1). The prevalence of Brucellosis among males was 22.9% (47/205). The study found a high prevalence of Brucellosis of 22.8% (62/272) among the participants aged 16–35 years. The prevalence of brucellosis was high, 19.5% (81/416) among patients who reported not having any knowledge about Brucellosis. Brucellosis was confirmed in 21.8% (39/179) of the patients who were Nilotic (Table 2).

**Table 2:** Prevalence of brucellosis among the study participants at Wau hospital.

Characteristic	Level	RBPT		SAT		C-Elisa		
		Negative (%)	Positive (%)	Negative (%)	Positive (%)	Negative (%)	Positive (%)	P-value
Sex	Female	73 (34.6)	138(65.4)	124(58.8)	87(41.2)	161(76.3)	50 (23.7)	0.853
	Male	61 (29.8)	144(70.2)	109(53.2)	96 (46.8)	158(77.1)	47 (22.9)	
Age	5–15	6 (23.1)	20 (76.9)	12 (46.2)	14 (53.8)	21 (80.8)	5 (19.2)	0.496
	16–35	87 (32.0)	185(68.0)	153(56.3)	119(43.8)	210(77.2)	62 (22.8)	
	36–60	36 (33.6)	71 (66.4)	61 (57.0)	46 (43.0)	78 (72.9)	29 (27.1)	
	>60	5 (45.5)	6 (54.5)	7 (63.6)	4 (36.4)	10 (90.9)	1 (9.1)	
Marital Status	Cohabiting	0 (0.0)	2 (100.0)	1 (50.0)	1 (50.0)	1 (50.0)	1 (50.0)	0.103
	Married	84 (33.1)	170(66.9)	141(55.5)	113(44.5)	187(73.6)	67 (26.4)	
	Single	50 (31.3)	110(68.8)	91 (56.9)	69 (43.1)	131(81.9)	29 (18.1)	
Education	No formal education (Illiterate)	39 (28.3)	99 (71.7)	72 (52.2)	66 (47.8)	103(74.6)	35 (25.4)	0.917
	Higher Education	22 (33.3)	44 (66.7)	43 (65.2)	23 (34.8)	52 (78.8)	14 (21.2)	
	Intermediate	27 (35.1)	50 (64.9)	42 (54.5)	35 (45.5)	60 (77.9)	17 (22.1)	
	Primary	26 (36.1)	46 (63.9)	42 (58.3)	30 (41.7)	57 (79.2)	15 (20.8)	
	Secondary	20 (31.7)	43 (68.3)	34 (54.0)	29 (46.0)	47 (74.6)	16 (25.4	
Religion	Others	2 (50.0)	2 (50.0)	3 (75.0)	1 (25.0)	3 (75.0)	1 (25.0)	0.997
	Christian	116 (32.6)	240(67.4)	202(56.7)	154(43.3)	273(76.7)	83 (23.3)	
	Muslim	16 (28.6)	40 (71.4)	28 (50.0)	28 (50.0)	43 (76.8)	13 (23.2)	
Occupation	Butcher	1 (33.3)	2 (66.7)	2 (66.7)	1 (33.3)	2 (66.7)	1 (33.3)	0.329
	Business/ Trader	7 (46.7)	8 (53.3)	8 (53.3)	7 (46.7)	14 (93.3)	1 (6.7)	
	Nurse/Midwife	30 (75.0)	1 (25.0)	3 (75.0)	1 (25.0)	4 (100.0)	0 (0.0)	
	Housewife	10 (28.6)	25 (71.4)	19 (54.3)	16 (45.7)	29 (82.9)	6 (17.1)	
	Student	36 (30.0)	84 (70.0)	70 (58.3)	50 (41.7)	95 (79.2)	25 (20.8)	
	Restaurant worker	10 (34.5)	19 (65.5)	15 (51.7)	14 (48.3)	24 (82.8)	5 (17.2)	
	Veterinary	1 (20.0)	4 (80.0)	4 (80.0)	1 (20.0)	3 (60.0)	2 (40.0)	
	Farmer	3 (33.3)	6 (66.7)	5 (55.6)	4 (44.4)	6 (66.7)	3 (33.3)	
	Jobless	14 (35.0)	26 (65.0)	28 (70.0)	12 (30.0)	26 (65.0)	14 (35.0)	
	Others	49 (31.4)	107(68.6)	79 (50.6)	77 (49.4)	116(74.4)	40 (25.6)	
Ethnic Group	Nilotic	53 (29.6)	126(70.4)	99 (55.3)	80 (44.7)	140(78.2)	39 (21.8)	0.028*
	Lou	16 (34.0)	31 (66.0)	28 (59.6)	19 (40.4)	40 (85.1)	7 (14.9)	
	Equatorian	8 (42.1)	11 (57.9)	13 (68.4)	6 (31.6)	15 (78.9)	4 (21.1)	
	Bantu	36 (38.7)	57 (61.3)	62 (66.7)	31 (33.3)	69 (74.2)	24 (25.8)	
	Arab	7 (29.2)	17 (70.8)	14 (58.3)	10 (41.7)	21 (87.5)	3 (12.5)	
	Fur	8 (19.0)	34 (81.0)	14 (33.3)	28 (66.7)	26 (61.9)	16 (38.1)	
	Falata	4 (50.0)	4 (50.0)	3 (37.5)	5 (62.5)	7 (87.5)	1 (12.5)	
	Others	2 (50.0)	2 (50.0)	0 (0.0)	4 (100.0)	1 (25.0)	3 (75.0)	
Know about Zoonotic diseases	No	121 (32.6)	250(67.4)	200(53.9)	171(46.1)	290(78.2)	81 (21.8)	0.040*
	Yes	13 (28.9)	32 (71.1)	33 (73.3)	12 (26.7)	29 (64.4)	16 (35.6)	

* Significant association.

%: Percentage.

Using c-ELISA, the study showed that 16.1% (67/416) of the patients who did not have any animal at home had Brucellosis. However, the rate of having a positive c-ELISA brucellosis test was higher, 28.6% (30/105) among patients who reported having animals at home compared to those who do not keep animals, 21.5% (67/311) (Table 3).

**Table 3:** Prevalence of brucellosis among participants who owned animals.

Characteristic	Level	Total	RBPT		SAT		C-Elisa	
			Negative (%)	Positive (%)	Negative (%)	Positive (%)	Negative (%)	Positive (%)
Own animals	No	311	102 (32.8)	209(67.2)	170(54.7)	141 (45.3)	244 (78.5)	67 (21.5)
	Yes	105	32 (30.5)	73 (69.5)	63 (60.0)	42 (40.0)	75 (71.4)	30 (28.6)
Type of animal	Large Ruminant	22	7 (31.8)	15 (68.2)	15 (68.2)	7 (31.8)	17 (77.3)	5 (22.7)
	Small ruminant	33	10 (30.3)	23 (69.7)	19 (57.6)	14 (42.4)	23 (69.7)	10 (30.3)
	Both	25	8 (32.0)	17 (68.0)	16 (64.0)	9 (36.0)	18 (72.0)	7 (28.0)
	Others	16	3 (18.8)	13 (81.3)	8 (50.0)	8 (50.0)	8 (50.0)	8 (50.0)
	NA	319	106 (33.2)	213(66.8)	175(54.9)	144 (45.1)	252 (79.0)	67 (21.0)

Amongst the patients who were confirmed to be having Brucellosis using c-ELISA, majority reported to the hospital with symptoms of fever and headache, 15.6% (65/416). Other patients with confirmed Brucellosis reported to the hospital with symptoms of; shivering 6.3% (26/416), fatigue 5.5% (23/416), joint pains 4.8% (20/416) and night sweats 1.9% (8/416). None of these disease symptoms were significantly associated with occurrence of Brucellosis among the study participants (P>0.05), (Table 4).

**Table 4:** Prevalence of brucellosis among patients and presenting clinical signs.

		Total	RBPT		SAT		C-Elisa	
			Negative (%)	Positive (%)	Negative (%)	Positive (%)	Negative (%)	Positive (%)
Fever	No	158	53(33.5)	105(66.5)	94 (59.5)	64 (40.5)	126 (79.7)	32 (20.3)
	Yes	258	81(31.4)	177(68.6)	139(53.9)	119 (46.1)	193 (74.8)	65 (25.2)
Headache	No	158	57(36.1)	101(63.9)	90 (57.0)	68 (43.0)	126 (79.7)	32 (20.3)
	Yes	258	77(29.8)	181(70.2)	143(55.4)	115 (44.6)	193 (74.8)	65 (25.2)
Shivering	No	301	103(34.2)	198(65.8)	172(57.1)	129 (42.9)	230 (76.4)	71 (23.6)
	Yes	115	31 (27.0)	84 (73.0)	61 (53.0)	54 (47.0)	89 (77.4)	26 (22.6)
Arthritis	No	347	116(33.4)	231(66.6)	196(56.5)	151 (43.5)	270 (77.8)	77 (22.2)
	Yes	69	18 (26.1)	51 (73.9)	37 (53.6)	32 (46.4)	49 (71.0)	20 (29.0)
Fatigue	No	343	112(32.7)	231(67.3)	195(56.9)	148 (43.1)	269 (78.4)	74 (21.6)
	Yes	73	22 (30.1)	51 (69.9)	38 (52.1)	35 (47.9)	50 (68.5)	23 (31.5)
Night sweating	No	387	123(31.8)	264(68.2)	221(57.1)	166 (42.9)	298 (77.0)	89 (23.0)
	Yes	29	11 (37.9)	18 (62.1)	12 (41.4)	17 (58.6)	21 (72.4)	8 (27.6)
Suffer from any	No	361	111(30.7)	250(69.3)	198(54.8)	163 (45.2)	279 (77.3)	82 (22.7)
other illness	Yes	52	23 (44.2)	29 (55.8)	33 (63.5)	19 (36.5)	39 (75.0)	13 (25.0)

The patients who reported consuming urine from the animals were twice more likely to have Brucellosis infection (OR: 2.94, 95%CI: 1.17–7.4, 0.02). The other predictors of Brucellosis infections among the study participants included, ethnicity and knowledge about zoonotic diseases were significant for brucellosis P < 0.05 (Table 5).

**Table 5:** Multivariate logistic regression of risk factors for brucellosis infection among study participants.

Factors	Characteristic	Unadjusted Odds ratio (95% CI)	Adjusted Odds ratio (95% C.I.)	Level of significance
Ethnic-Grouping	Others	1	1	-
	Nilotic	0.093 (0.009–0.918)	0.08 (0.01–0.77)	0.03
	Lou	0.058 (0.005–0.644)	0.05(0.004–0.53)	0.01
	Equatorian	0.089 (0.007–1.102)	0.07 (0.01–0.91)	0.04
	Bantu	0.116(0.012–1.168)	0.10(0.01–1.05)	0.06
	Arab	0.048(0.004–0.62)	0.04(0.003–0.54)	0.02
	Fur	0.205 (0.02–2.145)	0.17(0.02–1.82)	0.14
	Falata	0.048(0.002–1.040)	0.05 (0.002–1.04)	0.05
Knowledge about zoonotic diseases	No	0.506(0.262–0.978)	0.48 (0.24–0.96)	0.04
	Yes	1	1	-
Do you consume urine from animals	No	1	1	-
	Yes	2.616(1.068–6.411)	2.94 (1.17–7.4)	0.02

## Discussion

In this study, the prevalence of human brucellosis antibodies among patients attending an outpatient clinic at Wau referral Hospital was 23.3% this could be due to exposure or infection. This is higher than hospital based studies done in Uganda which reported prevalence of 13.3% and 17% [12, 13]. Other studies among febrile patients in, Dongla, Northern Sudan found prevalence of 15.3% [14], and 21.1% in Kenya [15]. However, Brucellosis prevalence in our study is lower than the reported prevalence of 40% in a study conducted in Libya [16]. The high prevalence of Brucellosis in this study could be attributed to the inadequate health and veterinary services in South Sudan. The methods of rearing animals, hygiene measures and limited awareness of communities on zoonotic diseases could be further contributing to the high Brucellosis prevalence observed in this study. A previous study found that the prevalence of human brucellosis is influenced by presence of brucellosis in domestic animals [17].

In this study, females and males had closely similar prevalence of Brucellosis, 23.7% and 22.9%, respectively. This could be due to consumption of infected animal products like raw milk, and meat that are from the same source. This is contrary to studies conducted in Egypt, Kuwait, Saudi Arabia and India [18] in which males had a higher rate of infection than females. In communities of South Sudan, females have equal opportunity of handling animals for example milking of cows, cutting raw meat, which potentially exposes them to the same level of risk of acquiring Brucellosis as their male counterparts. The prevalence of Brucellosis was higher among patients of age group 16-to-35 years, a finding similar to that of a previous study by El-Razik et al., 2007 [19]. In most cattle keeping communities, individuals of this age category play a central role in rearing animals in addition to performing other tasks such as milking cows and slaughtering of cattle. This could expose such individuals to the risk of acquiring Brucellosis and may be responsible for the high prevalence of the disease among this age group found in the current study.

In this study, the prevalence of Brucellosis was higher among Veterinarians and individuals who slaughter animals, 40% and 33.3% respectively. This was higher than that reported in Sudan (9.5%) and India (6.1%) (Ahmed and Sabah elkhier, 2015, [20]. This could be attributed to the inadequate veterinary services in the region, thus poor implementation and/or enforcement of preventive measures like hygiene in the abattoirs. This is evident from the findings of the current study in which there were only five (5) veterinarians in the whole region. Thus increasing the risk of contracting the disease among staff working in the animal slaughterhouses.

The patients who reported to be having knowledge on zoonotic diseases had low prevalence of Brucellosis. In addition, individuals who did not have any formal education had a high prevalence of Brucellosis in the current study. These findings could be attributed to the cultural practices of handling and consumption of animal products, which are common in pastoral communities. A study by Rafai, 2002 [6] showed that consumption of dairy products and delivery practices of animal products enhance spread of the disease.

Our current study showed that brucellosis prevalence was higher, among patients who reported having animals at home compared to those who do not keep animals. This could be attributed to the direct contact with infected animals or consumption of its infected products.

From multivariate analysis, consumption of urine increased the odds of getting Brucellosis among the study participants. In Addition, a study by Al-muneef et al., 2004 [21] showed that consumption of raw meat increased the risk of getting infected with Brucellosis. Individuals who reported to be consuming urine were twice more likely to have Brucellosis, a finding similar to that of a previous study by Lado et al., 2012[22]. Brucella bacteria are shed from the body mainly through urine, semen, milk and this could be responsible for the higher risk of contracting the disease among individuals who reported consuming urine, and raw milk in the current study [21, 23]. We further found that being a Nilotic increased the risk of having Brucellosis infection and this could attributed to the fact that majority of the Nilotic are mostly pastoralists in South Sudan.

### Limitation of the study

Instability in the region due to the civil war could have affected health-seeking behavior of the population especially as regards visiting the hospitals and thus we could have missed some patients who did not come to the hospital. The reported prevalence of Brucellosis potentially contains patients with no active disease as Brucella antibodies can persist in the body even after infection. There is a possibility that the approach used by our study could have missed out detecting sero-positivity among patients who may not have seroconverted at the time of data collection.

## Conclusion

Brucellosis is common among patients attending outpatient clinic in Wau hospital. Our study showed that consumption of infected animal products play major role in transmission of Brucellosis in the communities. There is need for Public awareness among community members and the healthcare professionals. This can be done through collaboration between veterinary and human health sectors of government.

## Supporting information

S1 Ethical Approval Approval from Makerere University Institutional Review Board. (PDF)

S2 Ethical Approval Approval from ministry of health (MOH), South Sudan to collect human and animal samples. (PDF)

S3 Ethical Approval Participants consents. (DOCX)

S4 Ethical Approval Import permit from Ministry of Agriculture, animal industry and fisheries (MAAIF) Uganda. (PDF)

S5 Ethical Approval Export permit from Ministry of livestock and fisheries industry, South Sudan. (PDF)

S1 Serological tests (DOC)

S1 Dataset Human dataset used for analysis. (SAV)

## Abbreviations

C-ELISA: Competitive Enzyme-Linked Immuno sorbent Assay
RBPT: Rose Bengal Plate Agglutination Test
CI: Confidence Interval
