# Supplementary material for: Prevalence of brucellosis among patients attending Wau Hospital, South Sudan
Source: PLoS One. 2018 Jun 27;13(6):e0199315. doi: 10.1371/journal.pone.0199315 (PMC6021100; doi:10.1371/journal.pone.0199315)
Supplement: S3 Ethical Approval — (DOCX) [file pone.0199315.s003.docx]

**PARTICIPANT CONSENT FORM**

**Makerere University**

**College of Veterinary Medicine Animal Resources and Biosecurity Department of Biosecurity, Ecosystems and Veterinary Public Health P.O. Box 7062 Kampala**

**Study title:** Brucellosis at Human-Domestic Animal interface in Greater Bahr el Ghazal States, South Sudan

**Site of research:** Greater Bahr el Ghazal, states, South Sudan

**Principle investigator:** Dr. Nuol Aywel Madut Yajj, Makerere University

College of veterinary medicine, Animal Resources and Biosecurity Department of preventive medicine & public health

**Purpose of the study:** Dr. Nuol Aywel Madut Yajj, Makerere University

College of veterinary medicine Animal Resources and Biosecurity (COVAB) is carrying out study to determine the sero-prevalence and risk factors associated with brucellosis in cattle and human in contact with animals in Greater Bahr elghazal states, South Sudan. Brucellosis is highly contagious bacterial disease and one of the world’s major zoonosis. It affects people and almost all domestic animals.

The objective of this study will therefore be to assess the people’s awareness, establish the sero-prevalence and associated risk factors for human and cattle brucellosis in Greater Bahr el ghazal states, South Sudan. The study and its findings will be beneficial to the entire community and all cattle farmers in the greater Bahr el ghazal states, and South Sudan at large, since it will form a basis for instituting control measures.

**How the study will be done:**

**Phase one**: After fully understanding this research, you may provide consent to participate in this study. If you do, a researcher will interview you and after this, blood samples will be taken from your cows and kept for brucellosis tests to be done later. After results are got from the laboratory, all those who participated will get their results and those whose animals test positive will be advised to participate willingly in phase two.

**Phase two:** Which include interview and screening for brucellosis by taking blood samples from you by medical personnel’s to be tested in the laboratory and according to the result we offer assistance for you in term of share treatment and advice in case your result is positive.

**Risk and discomforts:**

The risks of drawing blood from the Jugular vein or tail vein include temporary discomfort. The amount of blood removed will be too small to affect health of your animals. In case of phase two the blood collection will be in the hospital or nearest clinic by medical personnel’s.

**Confidentiality:**

Participant in research may involve a loss of privacy, but information about you and your animals will be kept as confidential as possible. People working on the study will only see this information. You will be identified only by code and your name will not appear on any study record.

**Benefits:**

There is no direct benefit to you for participating in this study. If your farm found positive, you will be referred to Veterinary Officers for advice.

**Cost/payment:**

There is no cost to you for participating in this study, and you will not be paid for participation.

**Alternatives to participation:**

Your participation in this study is voluntary.

**Use of the results:**

The findings from this study may be published in veterinary journal.

**Inquiries:**

For inquiries ask or call Dr. Nuol Aywel Madut Yajj, Tel: +256786986129

**Statement of consent:**

I………………………………………………..have understood what is going to be done, the risks, the benefits involved and my rights regarding this study. In the use of this information, my identity will be concealed. I understand that by signing this form, I indicate that I have been informed about the research study in which I am voluntarily agreeing to participate.

Signature or fingerprint of Participant:…………………………Date:…………………

Signature of the researcher or assistant:……………………… Date:………………….
