# Supplementary material for: Prevalence of brucellosis among patients attending Wau Hospital, South Sudan
Source: PLoS One. 2018 Jun 27;13(6):e0199315. doi: 10.1371/journal.pone.0199315 (PMC6021100; doi:10.1371/journal.pone.0199315)
Supplement: S1 Serological tests — (DOC) [file pone.0199315.s006.doc]

**Serological tests**

**Rose Bengal Plate Test (RBPT):**

Standardized buffered Rose Bengal stained antigen (provided by the Central Veterinary Research Laboratories, Soba, Sudan) was used to screen all obtained sera. The test was performed as follows: -

1. The serum samples and antigen were brought to room temperature (22±4°C).
2. Thirty-μL amount of each serum sample was placed on a white tile, enamel or plastic plate.
3. After shaking the antigen bottle well, but gently, an equal volume of 30 μL of the antigen was placed near each serum spot.
4. Immediately after the last drop of antigen was added to the plate, the serum and antigen were mixed thoroughly (using a clean glass or plastic rod for each test) to produce a circular or oval zone approximately 2 cm in diameter.
5. The mixtures were agitated gently for 4 minutes at ambient temperature on a rocker.
6. The agglutination was read immediately after the 4-minute shaking period was completed. Any visible reaction was considered to be positive.

RBPT was performed according to Alton et al., (1975).

**The serum agglutination test (SAT):**

Which historically has been the principle serological test used to detect brucellosis, measures agglutinating antibodies of the IgM, IgG1, IgG2, and IgA types. The SAT is relatively simple and easy to perform but it requires basic laboratory equipment. It can be used to detect acute infections, as antibodies of the IgM type usually appear first after infection and are more reactive in the SAT than antibodies of the IgG1 and IgG2 types. However, because the SAT may yield both false negative or false positive results. The antigen is a bacterial suspension in phenol saline i.e. NaCl 0.85%w/v and phenol at 0.5%v/v. antigens may be delivered in the concentrated state provided the dilution factor to be used is indicated on the bottle label. EDTA may be added to the antigen suspension to 5mM final test dilution to reduce the level of false-positive results. Subsequently the pH of 7.2 must be re-adjusted in the antigen suspension. The antigen shall be prepared without reference to the cell concentration, but its sensitivity must be standardized in such a way that the antigen produces either 50% agglutination with a final serum dilution of 1/600 to 1/1000 or 75% agglutination with a final serum dilution of 1/500 to 1/750. It may also be advisable to compare the reactivity of new and previously standardized batches of antigen using a panel of defined sera. Thaw test is performed either in tubes or in micro plates. The mixture of antigen and serum dilutions should be incubated for 16-24 hours at 37°C. If the test is carried out in micro plates, the incubation time can be shortened to 6 hours. At least three dilutions must be prepared for each serum in order to refute protozoan negative responders. Dilutions of suspect serum must be made in such a way that reading of the reaction at the positive limit is made in the median tube (or well for the micro plate method). Interpretation of agglutination results must be expressed in IU per ml. A serum containing 30 or more IU per ml is considered to be positive.

**The method:**

The antigen used for SAT was standardized concentrated antigen supplied by (central diagnostic laboratory, JICA), Makerere university, Kampala. The antigen was diluted to 1 to 12 using 5 ml phenol saline. The test was preformed as follows:

1. Eight test tubes were placed in row in a rack for each sample.
2. 0.8 ml of 5% NaCl solution was added to the first tube and 0.5 ml into each of the remaining seven tubes using 1 ml graduated pipette.
3. 0.2 ml of serum was added to the fist tube of each raw mixed well with the 5% NaCl by sucking and expelling gently to avoid producing bubbles.
4. 0.5 ml of mixes as transferred to the next tube, mixed well with the 5% NaCl, then 0.5 ml was transferred to the third tube.
5. Doubling the dilution was continued up to 8 tube then 0.5 ml from the last tube was discarded.
6. 0.5 ml of the antigen was added to each tube.
7. Control positive tubes containing equal amounts of antigen and known positive serum were included in the test.
8. Control negative tubes containing equal amounts of antigen and known negative serum were included in the test.
9. After shaking, the tubes were incubated at 37°C overnight.

The test was read by examine the tubes against aback background with light coming from behind the tubes appositive reaction is one in which the serum- antigen mixture is clear and agglutinated antigen appears at the bottom of the tube. Gentle shaking does not disrupt the floculi. This is a complete agglutination and is recorded as ++++. In partial agglutination serum-antigen mixture is partially clear and gentle shaking does not disrupt the floculi, this was recorded as +++ or ++. Some sedimentation as + and no clearing as negative reaction (Alton, 1975).

**Competitive Enzyme-Linked Immuno-sorbent Assay (C-ELISA):**

**Name and intended use**

IDEXX Brucellosis Serum is IDEXX’s enzyme immunoassay for the detection of Antibodies directed against Brucellosis in individual serum samples or pool of serum samples (Maximum 10) of bovine origin.

**Description and principle**

Micro plates are coated with brucellosis lipopolysaccharide (LPS). Samples to be tested are diluted and incubated in the wells. Upon incubation of the sample in the coated wells, Brucella specific antibodies from immune-complex with brucella LPS. After washing away unbound material, an anti-ruminant antibody enzyme Conjugate is added which binds to any immune-complex Brucella LPS-Antibody. Unbound Conjugate is washed away and enzyme Substrate (TMB) is added. In presence of the enzyme, the Substrate is oxidized and develops a blue compound becoming yellow after blocking. Subsequent color development is directly related to the amount of Antibody to Brucella present in the test Sample.

The result is obtained by comparing the sample Optical Density with the Positive Control mean Optical Density.

Reagents: (Store all reagents at 2- 8°C).

| Reagents |  | Volume | |
| --- | --- | --- | --- |
| 1 | Brucellosis LPS | 2 | 10 |
| 2 | Positive Control | 1 mL | 1 mL |
| 3 | Negative Control | 1 mL | 1 mL |
| 4a | (Anti-ruminant IgG HRPO) Conjugate Concentrate | 1.5 mL | 1.5 mL |
| 4b | Dilution Buffer N.1 | 120 mL | 120 mL |
| 5 | Dilution Buffer N.2 | 120 mL | 120 mL |
| A | TMB Substrate N.13 | 60 mL | 2 x 120 mL |
| B | Stop Solution N.3 | 60 mL | 120 mL |
| C | Wash Concentrate (20X) | 100 mL | 2 x 100 mL |

Materials Required:

1. Centrifuge (capacity 2000 x g)
2. Precision Micropipettes and Multi-dispensing micropipettes (reagents volumes)
3. Disposable pipette tips
4. Micoplate shaker
5. Distilled water or deionized water
6. Microplate washer (manual, semi-automatic or automatic system)
7. Microplate covers (lid, aluminum foil or adhesive)
8. 96-well Microplate reader equipped with 450nm filter

Preparation of Reagents

Wash Solution

The wash concentrate (20X) must be diluted 1:20 with distilled/deionized water before use (e.g. 15 ml of wash concentrate (20X) in 285 ml of distilled water). This solution is hereafter called “Wash Solution)”.

Conjugate

The Conjugate Concentrate must be:

- Diluted 1:100 in the Dilution Buffer N.1 when short protocol is used for sample incubation.
- Diluted 1:200 in the Dilution Buffer N.1 when overnight protocol is used for sample incubation.

Test Procedure

All reagents must be allowed to come to 18-26°C before use.

Reagents should be mixed by gentle swiring or vortexing. Use a separate pipette tip for each sample.

Controls may be dispensed anywhere on the micro plate.

Obtain coated micro plates and record the position of each sample on worksheet.

1. Dispense 190 μL of Dilution Buffer N.2 into each well.
2. Dispense 10 μL of Undiluted Negative Control into one appropriate well.
3. Dispense 10 μL of Undiluted Positive Control into one appropriate well.
4. Dispense 10 μL of Undiluted samples into one appropriate well.
5. Homogenize contents of the wells using a micro plate shaker.
6. Cover the micro plate (with a lid, aluminum foil or adhesive plate cover) and incubate for:

- Individual samples: 1 hour (± 5 min.) at 18- 26°C (short protocol) or 16- 24 hours at 18- 26°C (overnight protocol).
- Pool samples: 1 hour (± 5 min.) at 18- 26°C (short protocol).

1. Wash each well with approximately 300 μL of Wash Solution three times. Aspirate the liquid contents of all well after each wash. Following the final aspiration, firmly tap residual wash fluid from each micro plate onto absorbent material. Avoid micro plate drying between washes and prior to the addition of next reagent.
2. Dispense 100 μL of diluted Conjugate into each well.
3. Cover the micro plate (with lid, aluminum foil or adhesive plate cover) and incubate for 30 minutes (± 3 min.) at 18- 26°C.
4. Repeat step 7.
5. Dispense 100 μL of TMB-Substrate N.13 into each well.
6. Incubate 20 minutes (± 3 min.) at 18- 26°C in a dark place.
7. Dispense 100 μL of Stop Solution N.3 into each well. Shake the micro plate by gentle tapping. Wipe carefully the underside of the micro plate.
8. Blank the micro plate reader on air.
9. Measure and record Optical Densities values of samples and control at 450nm.
10. Calculate results.

Results

For the assay to be valid, the Positive Control Mean (PC
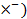
 must be greater than or equal to 0.350 optical Density (OD).

In addition, the ratio between the Positive Control mean (PC
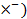
 and the Negative Control (NCA_450_) must be greater than or equal to 3.00.

Calculation

Calculation the sample to Positive (S/P) percentage for each sample:

Positive Control Mean Calculation for test sample


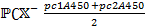
 S/P = 100 ×
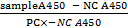


Interpretation of Results

Individual Serum

- Sample with S/P percentage less than or equal to 110 % are considered Negative for the presence of Brucella Antibodies.
- Sample with S/P percentage greater than 110 % and less than 120 % are considered Suspect.
- Samples with S/P percentage greater than or equal to 120% are considered Positive for the presence of Brucella Antibodies.

Pool of Sera

- Samples with S/p percentage less than 20% are considered Negative for Brucella Antibodies.
- Samples with S/P percentage greater than or equal to 20% are considered Positive for the presence of Brucella Antibodies.
